# Protein intake pattern over the day and its association with low total protein intake in Dutch community-dwelling older adults

**DOI:** 10.1017/S1368980020000026

**Published:** 2021-04

**Authors:** Teuni H Rooijackers, Marga C Ocké, Linda M Hengeveld, Marjolein Visser, Jolanda MA Boer

**Affiliations:** 1 Centre for Nutrition, Prevention and Health Services, National Institute for Public Health and the Environment (RIVM), 3721 MA Bilthoven, The Netherlands; 2 Division of Human Nutrition, Wageningen University & Research, Wageningen, The Netherlands; 3 Department of Health Sciences, Vrije Universiteit Amsterdam, Faculty of Science, Amsterdam Public Health Research Institute, Amsterdam, The Netherlands

**Keywords:** Protein intake pattern, Low protein intake, Older adults, Dutch National Food Consumption Survey

## Abstract

**Objective:**

Investigate protein intake patterns over the day and their association with total protein intake in older adults.

**Design:**

Cross-sectional study utilising the dietary data collected through two non-consecutive, dietary record-assisted 24-h recalls. Days with low protein intake (*n* 290) were defined using the RDA (<0·8 g protein/kg adjusted BW/d). For each day, the amount and proportion of protein ingested at every hour of the day and during morning, mid-day and evening hours was calculated. Amounts and proportions were compared between low and high protein intake days and related to total protein intake and risk of low protein intake.

**Setting:**

Community.

**Participants:**

739 Dutch community-dwelling adults ≥70 years.

**Results:**

The mean protein intake was 76·3 (sd 0·7) g/d. At each hour of the day, the *amount* of protein ingested was higher on days with a high protein intake than on days with a low protein intake and associated with a higher total protein intake. The proportion of protein ingested during morning hours was higher (22 *v*. 17 %, *P* < 0·0001) on days with a low protein intake, and a higher proportion of protein ingested during morning hours was associated with a lower total protein intake (*P* < 0·0001) and a higher odds of low protein intake (OR 1·04, 95 % CI 1·03, 1·06). For the proportion of protein intake during mid-day or evening hours, opposite but weaker associations were found.

**Conclusions:**

In this sample, timing of protein intake was associated with total protein intake. Additional studies need to clarify the importance of these findings to optimise protein intake.

A growing body of epidemiological and short-term metabolic studies indicate that protein intake above the dietary reference intake (DRI) benefits muscle mass, strength and function among older adults^(1)^. Furthermore, older persons with a lower protein intake are at increased risk of having mobility limitations^(2)^, a worse disability trajectory^(3)^ and of developing persistent protein-energy malnutrition (PEM)^(4)^.

The DRI of protein for healthy older adults is 0·8 g/kg BW/d^(5)^. National food consumption data indicate that the mean protein intake of Dutch community-dwelling older adults is equal or above the DRI, but variability is large. About 20 % of the community-dwelling older adults have an intake lower than the DRI^(6)^, and the prevalence of PEM in older adults varies from 7 % in home-living older adults to 33 % in hospitalised patients^(7–9)^.

There are indications that the *timing* of protein intake is important for total intake, but evidence is limited so far. In young adults, de Castro^(10)^ has found that eating a large proportion of protein in the morning is associated with a lower total protein intake. Studies in older adults are limited and show similar results^(11)^. Other previous studies often looked into meal occasions, while the exact time of the day of these meals was not incorporated. Additionally, any eating occasions reported outside the main meals were omitted or clustered into one category. Furthermore, they only investigated the proportion of protein eaten at different hours of the day and not the absolute amount of protein ingested (in grams)^(12–18)^.

It is important to look in more depth into the timing of protein intake, as to investigate whether it may inform optimal strategies for dietary interventions. Therefore, the present study aimed to describe the protein intake pattern of Dutch community-dwelling older adults and to evaluate whether total protein intake and the risk of low protein intake depend on the protein intake pattern over the day. Additionally, we investigated whether the pattern of protein intake over the day is related to possible determinants of PEM, that is, poor appetite, involuntary weight loss and a positive screening for PEM.

## Methods

### Study population

Data from the Dutch National Food Consumption Survey–Older Adults 2010–12 (DNFCS–Older Adults) were used. The rationale and methodology of this survey are described in detail elsewhere^(6)^. In short, of 2848 invited eligible men and women aged ≥70 years, 739 (26 %) participated in the study. Institutionalised older adults and those who were tube-fed or parenterally fed were not eligible. Additional exclusion criteria were having a high-intensity care package or being terminally ill. For practical reasons, older adults with impaired cognitive abilities and those with an inadequate command of the Dutch language were also excluded.

### Assessment of dietary intake

Dietary intake was assessed by means of two non-consecutive, dietary record-assisted 24-h recalls using a multiple pass approach. Subjects were interviewed at home by trained dieticians twice, with a mean interval of 4 weeks. Because impaired short-term memory is common in old age, subjects were asked to fill in a food diary on the day before the interview^(19)^. The completed diary was used as a memory aid during the 24-h recall the next day. The diary and 24-h recall covered the period from waking-up on the recall day until waking-up on the next day. Dieticians used a computer-controlled interview software (EPIC-Soft, IARC^©^)^(20–22)^ to directly enter the answers in the computer. Seven food consumption occasions were asked for, and information on all foods and drinks ingested were entered at one of the following: (i) before breakfast, (ii) breakfast, (iii) during morning, (iv) lunch, (v) during afternoon, (vi) dinner, (vii) during evening. For each occasion where food or drink was ingested, also the time (per full hour) of consumption was reported with ’07.00 hours’ indicating that the occasion started between 06.30 and 07.29 hours. Recalls were equally spread over all days of the week and the four seasons^(6)^. Both the subjects and the dieticians were unaware that the time of day of intake was being studied.

The consumed foods and drinks were converted into energy and nutrient intake using an extended version of the Dutch Food Composition Database (NEVO table 2011)^(23)^. Energy intake was expressed as kilojoules per day (kJ/d). Protein intake was expressed as grams per day (g/d), percentage of daily energy intake (En%), and as grams per kilogram adjusted BW per day (g/kg aBW/d) as suggested by Berner *et al.* ^(24)^. This was done to adjust for excessive BW (which comprises mostly of fat mass contributing little to the body protein turnover) among obese people as well as for insufficient protein availability to maintain muscle mass among underweight people. Using aBW may, therefore, be more sensible to detect the population at risk of low protein intake. aBW is the nearest BW that would place a participant with an undesirable BW in the healthy BMI range of 18·5–24·9 kg/m^2^ for adults aged ≤70 years, and of 22·0–27·0 kg/m^2^ for adults aged ≥71 years^(24)^. In total, 392 BW adjustments were made. If only measured BW was available and not body height (and BMI could, thus, not be calculated), actual weight was used (*n* 18). For twelve subjects, BW was missing, and therewith protein intake expressed as g/kg aBW/d. Recall days with a protein intake ≥0·8 g/kg aBW were labelled as days with a high protein intake, while those with intake <0·8 g/kg aBW were labelled as days with a low protein intake. The contribution of animal and vegetable protein and that of the three main food groups contributing to daily protein intake in Dutch older adults (‘meat, meat products and poultry’, ‘dairy products’ and ‘cereals and cereal products’^(6)^) were also calculated.

### Protein intake pattern over the day

We calculated the absolute amount of protein ingested for each hour of the day. Any occasion where food or drink was ingested was included. This provides, however, little information on the distribution of total protein intake over the day. Therefore, we also calculated the proportion of protein ingested during every hour of the day by dividing the amount of protein ingested at each hour of the day by total protein intake of that day.

### Screening for risk of undernutrition

Subjects were screened for (risk of) undernutrition during the first home visit with the Short Nutritional Assessment Questionnaire for 65^+^ (SNAQ^65+^)^(25)^. Short Nutritional Assessment Questionnaire for 65^+^ includes questions on appetite, unintentional weight loss and functional limitation. Appetite was assessed by asking whether a person experienced poor appetite in the past week (yes/no). Unintentional weight loss was assessed by asking whether a person unintentionally lost ≥4 kg within the past 6 months (yes/no). Functional limitation was assessed by asking whether a person could climb up and down a staircase of fifteen steps without stopping (yes/no). In addition, left mid-upper arm circumference was measured at the midpoint between the tip of the shoulder and the tip of the elbow. The following three groups were defined: (1) undernutrition (mid-upper arm circumference <25 cm or unintentional weight loss ≥4 kg in 6 months), (2) risk of undernutrition (poor appetite in the preceding week and difficulties climbing stairs) and (3) no undernutrition (others).

### Potential confounding variables

Information on age, sex, education, income, physical activity, type of housing and household composition was collected through an interviewer-administered general questionnaire during the first home visit. For the analyses, four categories of education were distinguished: primary education, lower vocational or advanced elementary education, intermediate vocational or higher secondary education, and higher vocational education or university. Income was categorised into low income (old age pension only) or middle/high income (old age pension with supplementary pension). Physical activity was based on responses to the Short QUestionnaire to ASsess Health enhancing physical activity (SQUASH)^(26)^. The activity level of subjects was classified based on the average number of days per week with at least 30 min of moderately intense physical activity. Distinguished categories were inactive (0 d), semi-active (0·5–4·5 d) or norm-active (≥5·0 d). Type of housing was aggregated into two categories: living fully independent (single-family dwelling, detached house, apartment, farm, flat) or living in a home especially intended for older adults (service flat, elderly commune, flat for elderly/pensioners/old people or living self-reliantly near a rest home). For the composition of the household, living alone was distinguished from living with partner, spouse or other person(s). Body height and weight were measured during one of the two home visits by trained dieticians, and BMI was calculated as weight in kilograms per height-squared in m^2^. Wake-up time was obtained by asking at what hour the subject woke up on the morning of each recall day.

### Statistical analysis

Participant characteristics were expressed as frequency and percentage for categorical data, and mean and standard deviation for continuous data. In the remainder of the analyses, recall days were used as the unit of observation.

Repeated-measures ANOVA was used to compare protein intake between hours of the day and between days with high and low protein intake. Hours that contributed <1 % to total protein intake over all recall days were excluded. As time of wake-up may affect the timing of protein intake, analyses were repeated while adjusting for wake-up time. In addition, the timing of protein intake was examined using time since wake-up. For these analyses, hours were defined as the number of hours being awake, with ’00.00 hours’ indicating that the occasion where food or drink was ingested started 0–59 min after the time of wake-up.

As a secondary analysis, differences in the timing of protein intake according to self-reported unintentional weight loss (≥4 kg in the past 6 months) were assessed using linear mixed models. A similar analysis was performed according to being screened as undernourished. To examine whether the timing of protein intake varied according to appetite, linear regression was used, using only the first 24-h recall. This was done because the question on poor appetite in the preceding week was solely asked during the first home visit.

To examine the association between the timing of protein intake and total protein intake, regression coefficients were calculated between the amount of protein ingested during every hour of the day (independent variable) and the total amount of protein ingested over that day (dependent variable) for every hour of the day. Since each subject contributed two observations, linear mixed models with an unstructured covariance matrix were used to account for within-person correlation. The association between the amount of protein ingested during every hour of the day and the risk of low protein intake was determined using mixed-effects modelling for logistic regression. Similar analyses were done with the proportion of protein ingested during every hour of the day as independent variable. Additional analyses were done expressing total protein intake in g/kg aBW, which yielded similar results. Therefore, these results are not presented.

Possible confounding variables were identified by ‘stepwise selection’. Effect modification by sex was checked by adding an interaction term with the proportion of protein intake to the model. Analyses were adjusted for age, sex, BMI and total energy intake (model 1) and additionally for wake-up time (model 2).

Four sensitivity analyses were conducted. First, we excluded non-typical days (due to, e.g., celebrations, illness or extreme tiredness, being very busy or away from home) and days on which a special diet was followed. Second, as some studies excluded occasions where <210 kJ was consumed^(27,28)^, analyses were repeated excluding these occasions to see whether this influenced the results. Third, possible influence of outliers was tested by removing the 1 % most extreme data points of both the total protein intake and amount or proportion of protein intake in the morning depending on the analysis. Among younger adults, dietary intake on weekdays tends to be lower and earlier in the day compared to weekend days^(27)^. Therefore, a fourth sensitivity analysis was conducted separately for weekdays (Monday through Thursday) and weekend days (Friday through Sunday). Friday was categorised as weekend day, since previous research has reported that intakes on Friday are more similar to those on Saturday and Sunday than they are to intakes on other weekdays^(27,28)^.

All analyses were performed using SAS software^®^, version 9.4, of the SAS System for Windows. The significance threshold was set at 0·05.

## Results

### Participant characteristics

The mean age of the study population was 77·1 (sd 5·2) years, and 49·5 % were women. Most were norm-active (78·6 %), lived in fully independent housing (88·5 %) and lived together with a partner or other person (61·9 %). The percentage being screened as undernourished was 9·6 % (Table 1).

**Table 1:** Characteristics of Dutch community-dwelling older adults aged ≥70 years (Dutch National Food Consumption Survey–Older Adults 2010–12)*

	Total population (*n* 739)		
	*n*		%
Age (years)			
Mean		77·1	
sd		5·2	
Women	366		49·5
BMI (kg/m^2^) (*n* 727)			
Mean		27·4	
sd		3·8	
Education level (*n* 736)			
Primary education	128		17·4
Lower vocational or advanced elementary education	280		38·0
Intermediate vocational or higher secondary education	161		21·9
Higher vocational education or university	167		22·7
Physical activity (*n* 738)			
Inactive	32		4·3
Semi-active	126		17·1
Norm-active	580		78·6
Living fully independent	654		88·5
Living together (*n* 738)	457		61·9
Income status (*n* 730)			
Low	83		11·4
Middle/high	647		88·6
SNAQ^65+^ (*n* 732)			
Undernourished	70		9·6
Risk of undernutrition	5		0·7
Mid-upper arm circumference < 25 cm	27		3·7
Unintentional weight loss ≥ 4 kg in the past 6 months (*n* 738)	52		7·1
Poor appetite in the past week (*n* 738)	32		4·3
Difficulties climbing staircase (*n* 738)	71		9·6

SNAQ^65+^, Short Nutritional Assessment Questionnaire for 65+.

* Data are presented as number of participants and percentages (all categorical variables); mean values and standard deviations (all continuous variables).

### Total protein intake

The mean dietary protein intake was 76·3 (se 0·7) g/d, 1·05 (se 0·01) g/kg aBW/d and 15·7 (se 0·1) En% (Table 2). Animal protein, especially from meat and dairy products, contributed most to total protein intake (62·0 (se 0·4) %). On 19·6 % (*n* 290) of all recall days, the reported protein intake was low, that is, <0·8 g/kg aBW. The mean protein intake on these days was 51·1 (se 1·1) g as compared to 82·8 (se 0·6) g on days with a high protein intake. Also, the contribution of protein to total energy intake was lower (13·2 (se 0·2) *v*. 16·4 (se 0·1) %, respectively). The difference in animal protein intake between days with low and days with high protein intake (27·8 (se 1·0) *v*. 53·6 (se 0·5) g/d, *P* < 0·0001) was larger than the difference in vegetable protein intake (23·8 (se 0·5) *v*. 29·0 (se 0·3) g/d, *P* < 0·0001). This resulted in a larger proportion of vegetable protein on days with a low protein intake compared to days with a high protein intake (45·8 (se 0·7) *v*. 36·0 (se 0·4) %, *P* < 0·0001).

**Table 2:** Mean energy and protein intake of Dutch community-dwelling older adults aged ≥70 years for all recall days and separately for days with a low protein intake and days with a high protein intake (Dutch National Food Consumption Survey–Older Adults 2010–12)

		All recall days (*n* 1478)		Days with a high protein intake (*n* 1164)		Days with a low protein intake† (*n* 290)	
		Mean	se	Mean	se	Mean	se
Energy intake	kJ/d	8257	73	8619	70	6830	115*
Protein intake	g/d	76·3	0·7	82·8	0·6	51·1	1·1*
	g/kg aBW/d‡	1·05	0·01	1·15	0·01	0·68	0·02*
	En%	15·7	0·1	16·4	0·1	13·2	0·2*
Protein sources							
Vegetable protein	g/d	27·9	0·3	29·0	0·3	23·8	0·5*
	% of total protein	37·9	0·4	36·0	0·4	45·8	0·7*
Animal protein	g/d	48·4	0·6	53·6	0·5	27·8	1·0*
	% of total protein	62·0	0·4	63·9	0·4	54·1	0·7*
Protein-rich food groups							
Meat, meat products and poultry	g/d	86·1	1·8	95·1	1·9	49·2	3·6*
	% of total protein	26·5	0·5	28·0	0·5	20·3	1·0*
Dairy products§	g/d	350·0	7·6	369·3	7·8	273·9	12·1*
	% of total protein	25·0	0·4	24·8	0·4	26·1	0·8*
Cereals and cereal products	g/d	162·8	2·7	169·1	2·9	140·0	5·2*
	% of total protein	20·9	0·3	19·8	0·3	25·4	0·5*

aBW, adjusted body weight; En%, percentage of energy intake.

† Days with a low protein intake represent days with protein intake <0·8 g/kg aBW.

‡ Grams of protein per kilogram BW calculated after adjustment to the nearest weight that would place the subject in the healthy BMI range (18·5–24·9 kg/m^2^ for adults ≤70 years; 22–27 kg/m^2^ for adults aged ≥71 years). Missing for *n* 24 recall days.

§ Dairy products represent milk and milk products and cheese.

* *P* ≤ 0·05 for days with a low *v*. days with a high protein intake.

### Protein intake pattern over the day

**Fig. 1:**
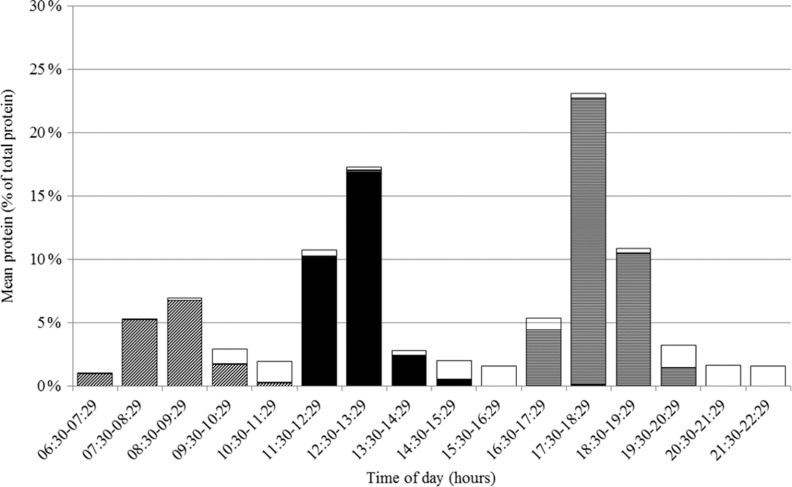
Proportion of total protein intake in Dutch community-dwelling older adults aged ≥70 years across time of the day and specified for breakfast (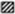), lunch (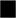), dinner (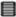) and in between meal (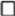) occasions (Dutch National Food Consumption Survey–Older Adults 2010–12)

The pattern of protein intake over the day is presented in Fig. 1. Protein intake, expressed as the proportion of total protein intake, differed across hours of the day (*F* 371·6, *P* < 0·0001) with peaks between 08.30 and 09.29 hours (mostly breakfast), 12.30 and 13.29 hours (mostly lunch) and 17.30 and 18.29 hours (mostly dinner). Adjusting for wake-up time did not influence the proportions nor the differences in proportions across hours of the day. The proportion of protein intake also differed across hours of being awake (*F* 159·3, *P* < 0·0001) with peaks between 00.00–00.59, 05.00–05.59 and 10.00–10.59 hours after waking up (Supplemental Fig. S1). Excluding non-typical days, occasions providing <210 kJ energy or outliers did not influence the protein intake patterns. On weekdays, protein intake was earlier in the day than on weekend days, but total protein intake was similar (75·5 (se 0·9) *v*. 77·2 (se 1·0) g/d, *P* = 0·13).

### Differences in protein intake pattern between days with low and high protein intake

**Fig. 2:**
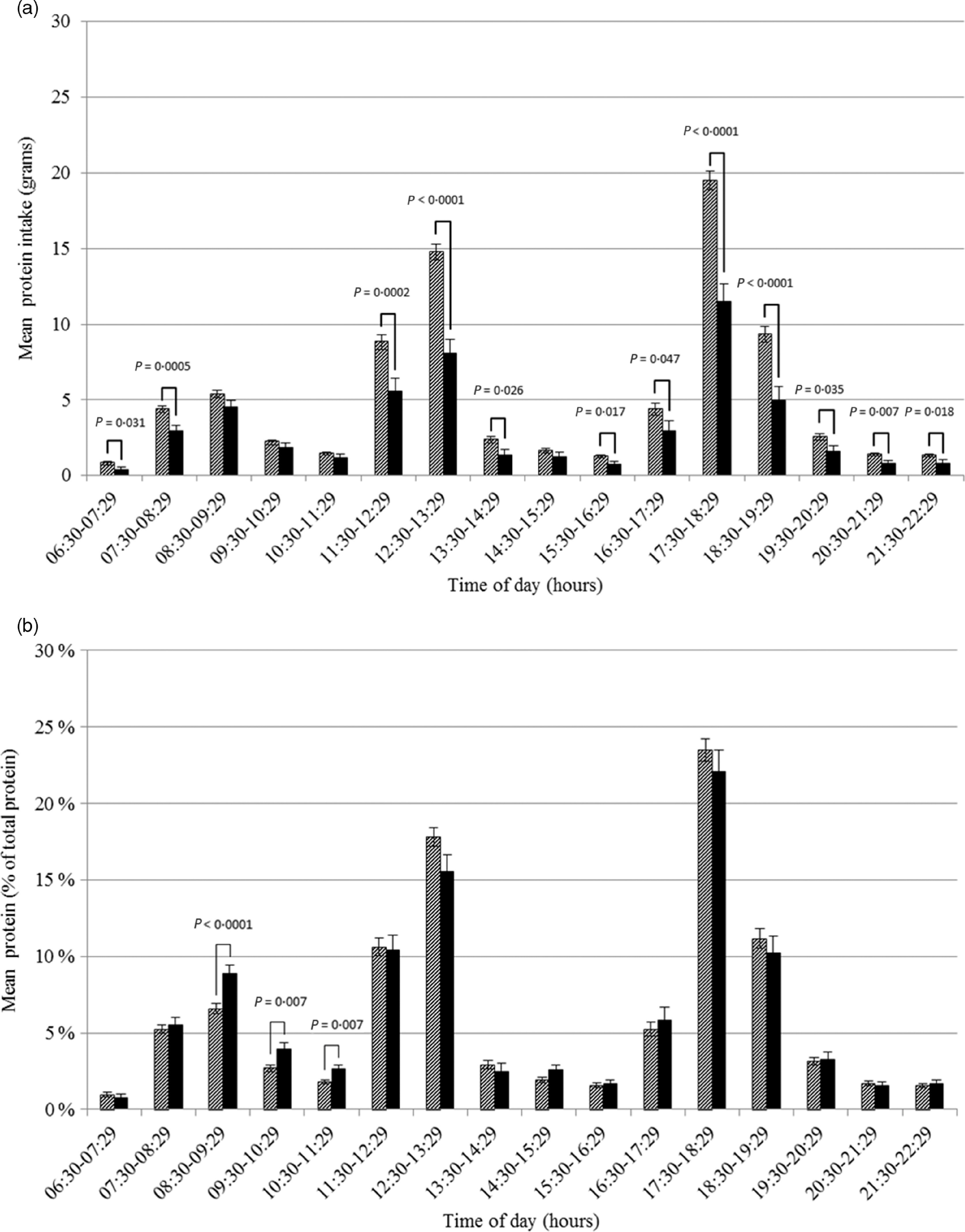
Amount (a) and proportion (b) of total protein intake in Dutch community-dwelling older adults aged ≥70 years across time of the day and stratified by low protein intake (Dutch National Food Consumption Survey–Older Adults 2010–12). 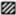, days with a high protein intake; 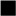, days with a low protein intake

At each hour of the day, absolute protein intake was lower on days with a low protein intake than on days with a high protein intake. Only at four time-points the difference was not statistically significant (Fig. 2(a)). The differences were largest (>3 g) for intake between 11.30–12.29, 12.30–13.29, 17.30–18.29 and 18.30–19.29 hours. Relatively (as a proportion of total protein intake), a significantly higher proportion of protein was ingested between 08.30–09.29, 09.30–10.29 and 10.30–11.29 hours on days with a low protein intake compared to days with a high protein intake (Fig. 2(b)). No statistically significant differences were observed for hours later in the day. Similar results were found when using time since wake-up instead of actual time (00.00–00.59 hours after wake-up, Δ_mean_ 1·82 %, *P* = 0·003; 01.00–01.59 hours after wake-up, Δ_mean_ 1·44 %, *P* = 0·022).

### Differences in protein intake pattern according to appetite, unintentional weight loss and undernutrition

**Fig. 3:**
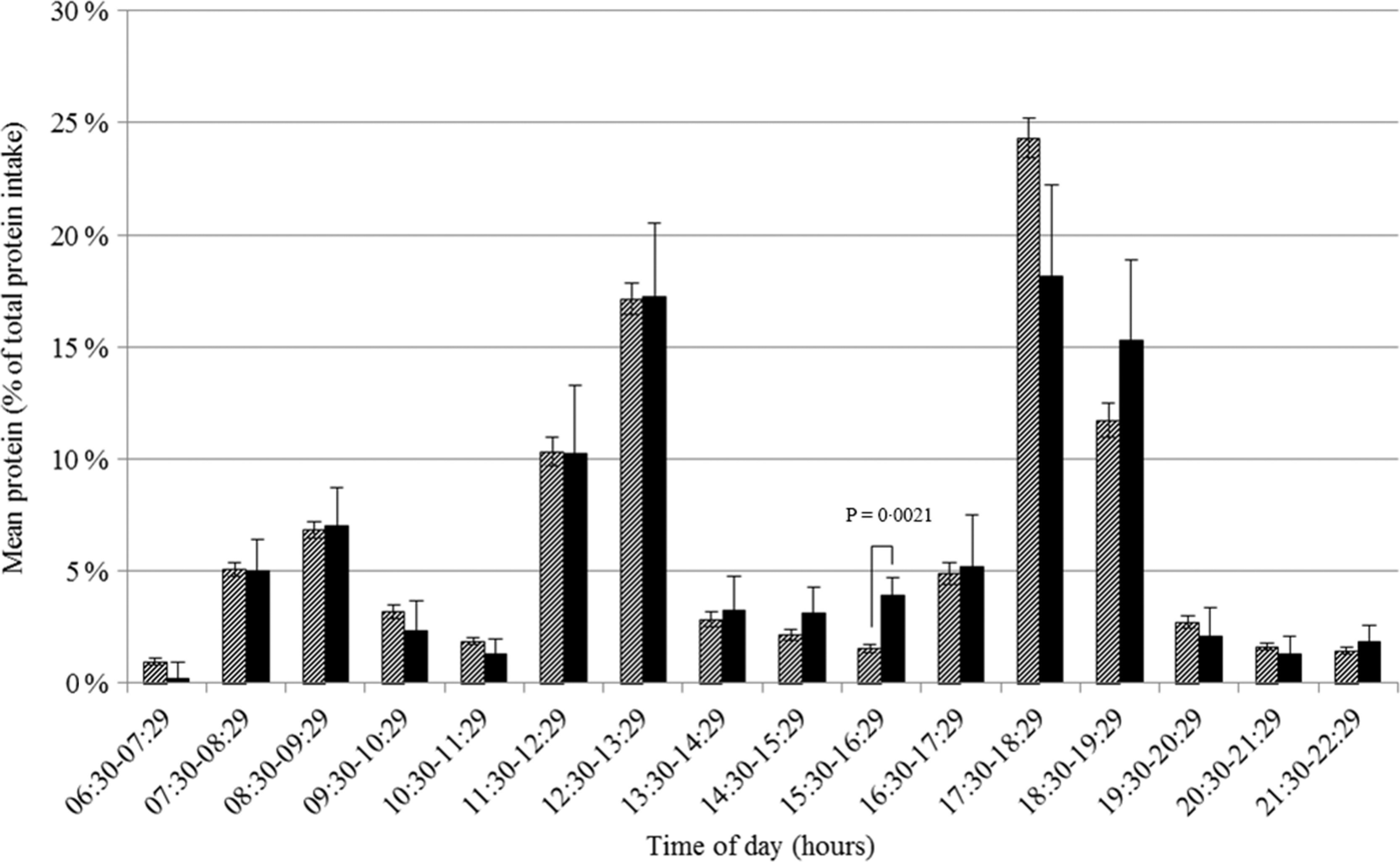
Proportion of total protein intake in Dutch community-dwelling older adults aged ≥70 years across time of the day and stratified by loss of appetite in the past week (Dutch National Food Consumption Survey–Older Adults 2010–12). 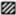, no appetite loss; 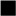, appetite loss

Protein intake was somewhat lower on days of subjects reporting a poor appetite than on days of subjects not reporting a poor appetite (71·1 (se 4·1) *v*. 77·3 (se 0·9) g), but this difference was not statistically significant (*P* = 0·15). There was no difference in the protein intake pattern over the day between these two groups (Fig. 3). Furthermore, no differences in the protein intake pattern were found according to reported unintentional weight loss. For 2 h of the day, the proportion of protein intake on days of subjects being screened as undernourished differed significantly from the proportion on days of subjects not being screened as undernourished. However, for 17.30–18.29 hours the proportion was higher (27·7 (se 2·3) *v*. 22·9 (se 0·7) %, *P* = 0·048) and for 18.30–19.29 hours the proportion was lower (11·6 (se 0·6) *v*. 6·2 (2·0) %, *P* = 0·0089), suggesting no clear shift in the timing of protein intake.

### Association between the protein intake pattern over the day and total protein intake

For all but one hour of the day, the amount of protein ingested that hour was associated with total protein intake. In contrast, there was a significant association between the proportion of protein intake and total protein intake only for several morning hours (08.30–09.29 hours, *β*_1_ –0·16, *P* < 0·0001; 09.30–10.29 hours, *β*_1_ –0·13, *P* = 0·030; 10.30–11.29 hours, *β*_1_ –0·23, *P* = 0·012). Therefore, further analyses focused on morning protein intakes (between 06.30 and 11.29 hours pooled together). For the other remaining hours, similar timeframes were defined (11.30–16.29 and 16.30–22.29 hours), representing mid-day and evening.

The *amount* of protein intake in the morning, mid-day and evening was lower on days with a low protein intake as compared to days with a high protein intake (11·4 (se 0·4) *v*. 14·1 (se 0·3) g, 16·9 (se 0·9) *v*. 28·9 (se 0·5) g and 22·6 (se 0·9) *v*. 38·5 (se 0·5) g, respectively; all *P* < 0·0001).

**Fig. 4:**
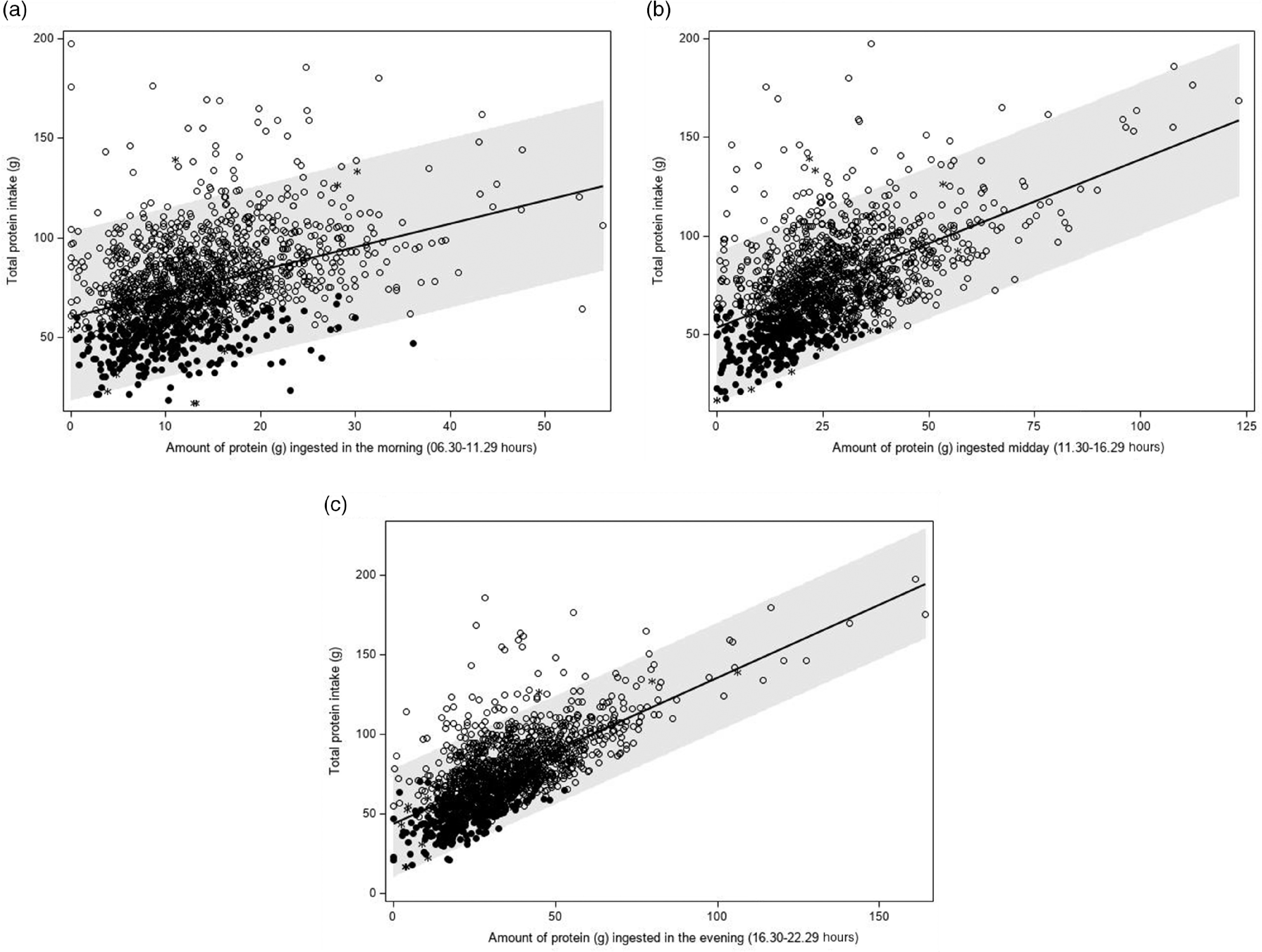
Association between the amount of protein ingested in the morning (a: 06.30–11.29 hours), mid-day (b: 11.30–16.29 hours) or evening (c: 16.30–22.29 hours) and total protein intake (g/d) (Dutch National Food Consumption Survey–Older Adults 2010–12). 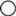, day with a high protein intake; 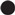, day with a low protein intake; 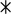, non-labelled day

A higher *amount* of protein intake in the morning, mid-day and evening were all associated with a higher total protein intake (*β*_1_ 1·11, 0·86 and 0·90 g, respectively; all *P* < 0·0001), also after adjusting for age, sex, BMI and total energy intake (*β*_1_ 0·46, 0·55, 0·65 g; *P* < 0·0001) (Table 3 and Fig. 4). The sensitivity analyses yielded comparable results. When time was expressed in hours being awake, regression coefficients were smaller.

**Table 3:** Association between the amount of protein (g) ingested in the morning (06.30–11.29 hours), mid-day (11.30–16.29 hours) and evening (16.30–22.29 hours) and total protein intake and risk of low protein intake (Dutch National Food Consumption Survey–Older Adults 2010–12)

	Total protein (g/d) (*n* 1478)						Low protein intake day (*n* 1454)					
	06.30–11.29 hours		11.30–16.29 hours		16.30–22.29 hours		06.30–11.29 hours		11.30–16.29 hours		16.30–22.29 hours	
	*β*_1_ *	95 % CI	*β*_1_ *	95 % CI	*β*_1_ *	95 % CI	OR†	95 % CI	OR†	95 % CI	OR†	95 % CI
Absolute time-of-day												
Crude model	1·11	0·96, 1·26	0·86	0·79, 0·93	0·90	0·86, 0·95	0·92	0·90, 0·94	0·90	0·89, 0·92	0·90	0·88, 0·91
Model 1‡	0·46	0·34, 0·58	0·55	0·49, 0·61	0·65	0·60, 0·70	0·95	0·93, 0·98	0·92	0·90, 0·93	0·91	0·89, 0·93
Model 2§	0·47	0·35, 0·60	0·56	0·50, 0·61	0·65	0·61, 0·70	0·95	0·93, 0·98	0·92	0·90, 0·93	0·91	0·89, 0·93
Sensitivity analyses												
Exclusion 1‖	0·46	0·33, 0·59	0·57	0·51, 0·62	0·65	0·60, 0·70	0·95	0·93, 0·98	0·92	0·90, 0·93	0·91	0·90, 0·93
Exclusion 2¶	0·45	0·31, 0·60	0·55	0·48, 0·61	0·65	0·60, 0·71	0·95	0·92, 0·98	0·92	0·90, 0·94	0·91	0·89, 0·93
Exclusion 3**	0·46	0·34, 0·58	0·55	0·49, 0·61	0·65	0·60, 0·70	0·95	0·93, 0·98	0·92	0·90, 0·93	0·91	0·89, 0·93
Exclusion 4††	0·48	0·36, 0·60	0·51	0·45, 0·57	0·61	0·56, 0·66	0·95	0·92, 0·97	0·92	0·90, 0·93	0·90	0·88, 0·92
Weekday	0·38	0·22, 0·54	0·53	0·46, 0·61	0·64	0·58, 0·70	0·95	0·91, 0·98	0·93	0·91, 0·95	0·91	0·89, 0·93
Weekend day	0·56	0·38, 0·74	0·56	0·47, 0·64	0·64	0·56, 0·71	0·96	0·92, 1·00	0·90	0·88, 0·93	0·92	0·89, 0·94
Time since wake-up	0–4 h		5–9 h		10–14 h		0–4 h		5–9 h		10–14 h	
Crude model	0·49	0·41, 0·56	0·54	0·49, 0·59	0·56	0·51, 0·62	0·95	0·94, 0·96	0·96	0·95, 0·97	0·95	0·94, 0·96
Model 1	0·28	0·23, 0·34	0·33	0·28, 0·37	0·32	0·28, 0·37	0·96	0·94, 0·97	0·96	0·95, 0·98	0·97	0·96, 0·98

*β*_1_, slope.

* *β* for a 1 % higher amount of protein intake.

† OR for a 1 % increase in the amount of protein ingested.

‡ Model 1: adjusted for age + sex + BMI + energy intake.

§ Model 2: adjusted for age + sex + BMI + energy intake + wake-up time.

‖ Exclusion 1: model 1 excluding non-typical days.

¶ Exclusion 2: model 1 excluding special diets.

** Exclusion 3: model 1 excluding occasions with energy intake <210 kJ.

†† Exclusion 4: model 1 excluding 1 % most extreme data points of the amount of protein intake per time period and total protein intake.

The *proportion* of protein intake ingested in the morning was higher on days with a low protein intake than on days with a high protein intake (22·0 (se 0·6) *v*. 17·2 (se 0·3) %, *P* < 0·0001). The proportion of protein intake mid-day and evening was lower on days with a low protein intake (32·3 (se 0·9) *v*. 34·9 (se 0·5) g, *P* = 0·006; and 44·4 (se 0·9) *v*. 46·3 (se 0·5) g, respectively, *P* = 0·048).

**Fig. 5:**
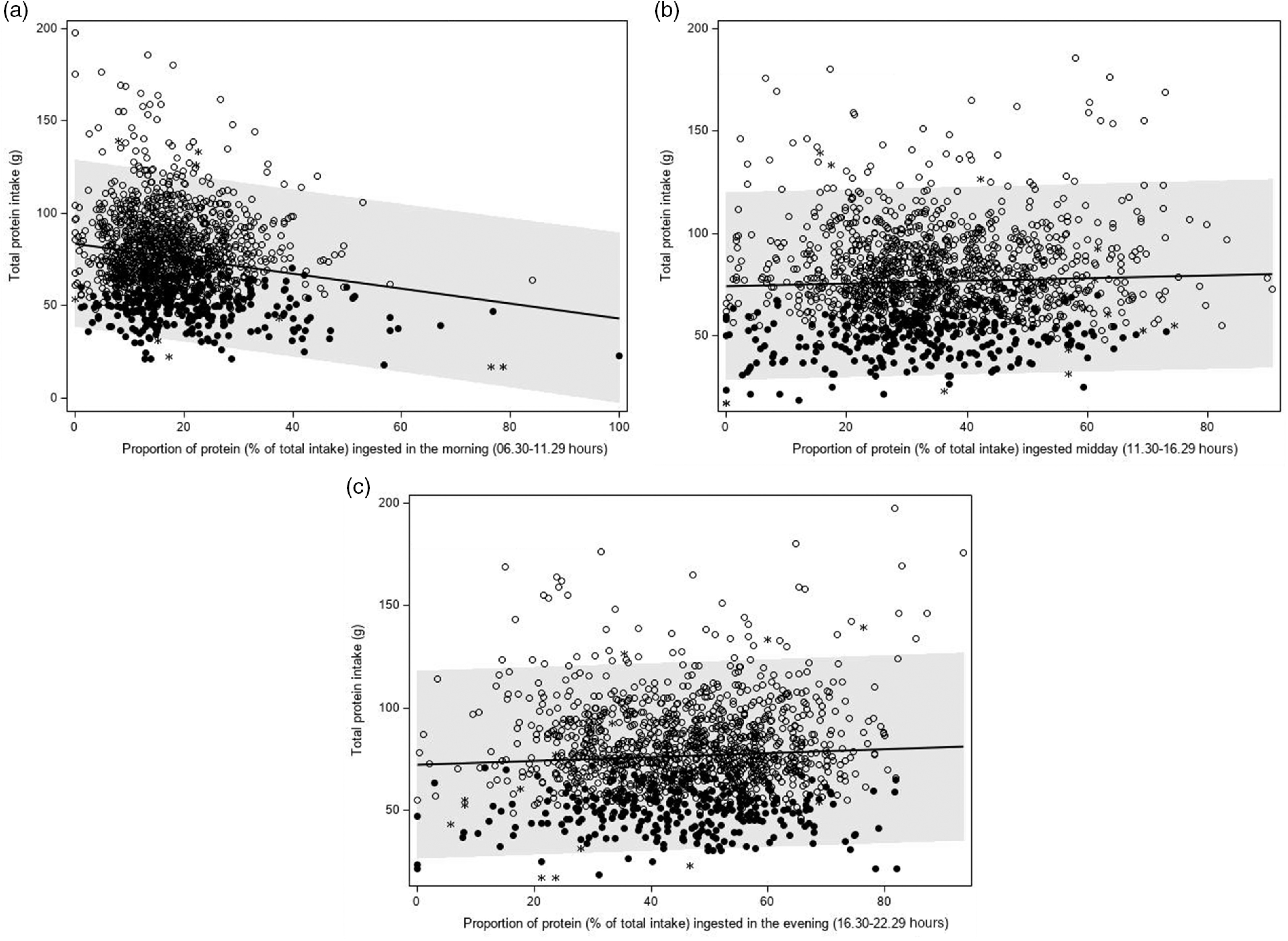
Association between the proportion of protein ingested in the morning (a: 06.30–11.29 hours), mid-day (b: 11.30–16.29 hours) or evening (c: 16.30–22.29 hours) and total protein intake (g/d) (Dutch National Food Consumption Survey–Older Adults 2010–12). 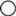, day with a high protein intake; 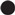, day with a low protein intake; 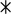, non-labelled day

A higher *proportion* of protein intake in the morning was significantly associated with a lower total protein intake (*β*_1_ –0·58 g, *P* < 0·0001) (Table 4 and Fig. 5(a)). A higher *proportion* of protein intake mid-day or evening was associated with a higher total protein intake (*β*_1_ 0·09 g, *P* = 0·035 and *β*_1_ 0·14 g, *P* = 0·0003, respectively), but regression coefficients were smaller than those observed for the proportion of protein ingested in the morning (Table 4, Fig. 5(b) and (c)). The regression coefficients decreased slightly after adjusting for age, sex, BMI and total energy intake and were quite similar for weekdays and weekend days. Further adjusting for wake-up time did not alter the abovementioned findings. When time was expressed in hours being awake, regression coefficients were smaller. Excluding non-typical days, occasions providing <210 kJ energy and outliers had little effect on the regression coefficients.

**Table 4:** Association between the proportion of protein (g) ingested in the morning (06.30–11.29 hours), mid-day (11.30–16.29 hours) and evening (16.30–22.29 hours) and total protein intake and risk of low protein intake (Dutch National Food Consumption Survey–Older Adults 2010–12)

	Total protein (g/d) (*n* 1478)						Low protein intake day (*n* 1454)					
	06.30–11.29 hours		11.30–16.29 hours		16.30–22.29 hours		06.30–11.29 hours		11.30–16.29 hours		16.30–22.29 hours	
	*β*_1_ *	95 % CI	*β*_1_ *	95 % CI	*β*_1_ *	95 % CI	OR†	95 % CI	OR†	95 % CI	OR†	95 % CI
Absolute time-of-day												
Crude model	–0·58	–0·70, –0·46	0·09	0·01, 0·16	0·14	0·07, 0·22	1·04	1·03, 1·06	0·99	0·98, 1·00	0·99	0·99, 1·00
Model 1‡	–0·37	–0·46, –0·28	0·07	0·01, 0·13	0·09	0·03, 0·15	1·04	1·03, 1·06	0·99	0·98, 1·00	1·00	0·99, 1·01
Model 2§	–0·37	–0·47, –0·29	0·07	0·02, 0·13	0·09	0·03, 0·15	1·04	1·03, 1·06	0·99	0·98, 1·00	1·00	0·99, 1·01
Sensitivity analyses												
Exclusion 1‖	–0·39	–0·49, –0·30	0·08	0·02, 0·15	0·08	0·02, 0·14	1·04	1·03, 1·06	0·99	0·97, 1·00	1·00	0·99, 1·01
Exclusion 2¶	–0·45	–0·56, –0·34	0·07	0·00, 0·13	0·10	0·03, 0·16	1·05	1·03, 1·07	0·99	0·98, 1·00	1·00	0·98, 1·01
Exclusion 3**	–0·37	–0·46, –0·28	0·07	0·01, 0·13	0·09	0·03, 0·15	1·04	1·03, 1·06	0·99	0·98, 1·00	1·00	0·99, 1·01
Exclusion 4††	–0·37	–0·46, –0·28	0·00	–0·06, 0·06	0·03	–0·03, 0·08	1·05	1·03, 1·06	0·99	0·98, 1·00	1·00	0·99, 1·01
Weekday	–0·31	–0·42, –0·20	0·04	–0·03, 0·12	0·10	0·03, 0·18	1·04	1·01, 1·06	0·99	0·98, 1·01	0·99	0·98, 1·01
Weekend day	–0·34	–0·48, –0·20	0·10	0·02, 0·19	0·03	–0·06, 0·12	1·05	1·02, 1·07	0·98	0·96, 0·99	1·00	0·99, 1·02
Time since wake-up	0–4 h		5–9 h		10–14 h		0–4 h		5–9 h		10–14 h	
Crude model	–0·16	–0·22, –0·09	0·06	0·00, 0·11	0·06	0·01, 0·12	1·01	1·00, 1·02	1·00	0·99, 1·00	1·00	0·99, 1·00
Model 1	–0·05	–0·10, –0·01	0·04	0·00, 0·08	0·01	–0·03, 0·05	1·00	0·99, 1·01	1·00	0·99, 1·00	1·00	0·99, 1·01

*β*_1_, slope.

* *β* for a 1 % higher amount of protein intake.

† OR for a 1 % increase in the amount of protein ingested.

‡ Model 1: adjusted for age + sex + BMI + energy intake.

§ Model 2: adjusted for age + sex + BMI + energy intake + wake-up time.

‖ Exclusion 1: model 1 excluding non-typical days.

¶ Exclusion 2: model 1 excluding special diets.

** Exclusion 3: model 1 excluding occasions with energy intake <210 kJ.

†† Exclusion 4: model 1 excluding 1 % most extreme data points of the amount of protein intake per time period and total protein intake.

### Association between protein pattern and the odds of low protein intake

An increase in the *amount* of protein intake in the morning, mid-day or evening resulted in a lower odds of low protein intake (Table 3). An increase in the *proportion* of protein intake in the morning, however, was significantly associated with a higher odds of low protein intake (OR per 1 % increase in the proportion of protein intake in the morning: 1·04, 95 % CI 1·03, 1·06) (Table 4). For protein intake mid-day, an opposite but weaker association was observed (OR 0·99, 95 % CI 0·98, 1·00 after adjusting for age, sex, BMI and energy intake). For the evening hours, the association was not statistically significant after adjustment (OR 1·00, 95 % CI 0·99, 1·01). In the sensitivity analyses, similar results were obtained.

## Discussion

The present study provides detailed information on the protein intake patterns over the day in Dutch community-dwelling older adults. Protein intake varied considerably over the day, and the distribution of protein intake over the day differed between days with a low protein intake and days with a high protein intake. The *amount* of protein ingested in the morning, mid-day or evening was lower on days with a low protein intake. In addition, a higher *amount* of protein intake in all three parts of the day was associated with a higher total protein intake and a lower risk of low protein intake. In contrast, the morning hours accounted for a larger *proportion* of total protein intake on days with a low protein intake, and a higher *proportion* of protein in the morning was associated with a lower total protein intake and a higher risk of low protein intake. Associations for the *proportion* of protein ingested mid-day or evening were much weaker. Associations were insensitive to adjustment for wake-up time, exclusion of non-typical days, outliers and occasions providing <210 kJ, and were consistent across weekdays and weekend days. These results suggest that the timing of protein intake may affect total protein intake.

Our findings in older persons extend those of de Castro^(10)^ who found that the proportion of protein intake in the morning was negatively associated with total protein intake among 867 men and women with a mean age of 36 years. To the best of our knowledge, there is only one other study that addressed the protein intake pattern in relation to total protein intake in older adults. Also, in that study, morning eating contributed more to total protein intake in the group of old (85+) men and women defined as having a low protein intake using the same cut-off values as we did^(11)^. de Castro^(10)^ postulated that a higher contribution of morning meals to protein intake might produce greater satiety than later in the day. This hypothesis is supported by Leidy *et al*.^(17)^ who found that a breakfast high in protein (1·4 g/kg BW/d) led to greater overall fullness compared to a high-protein lunch or dinner in nine men (mean age 48 years) during energy restriction. In contrast, Bollwein *et al*.^(15)^ have reported that among community-dwelling older adults (≥75 years), frail subjects ingested significantly less protein at breakfast (absolute intake), but more protein at lunch, than pre-frail and non-frail older adults. However, no differences in total protein intake were found. This indicates that morning protein intakes contributed less to total protein intake in frail compared to pre-frail and non-frail older adults. The time at which the meals were consumed was not reported. Therefore, it is not possible to determine whether differences in the timing of meals might have played a role.

A higher *amount* of protein in the morning is associated with a higher total protein intake and a lower risk of low protein intake even when adjusting for total energy intake. Similar positive associations were observed for the amount of protein ingested mid-day or evening. Based on these associations, one may hypothesise that increasing protein intake at any time of day may positively affect total protein intake. A higher intake at either time of day may, however, probably just reflect a higher food consumption and does not necessarily provide information on the distribution of protein intake over the day. The association between the *proportion* of energy intake in the morning and total protein intake observed in our study was stronger than the association between the proportion of energy ingested mid-day and that in the evening. Based on these associations and the supporting evidence described above, one may hypothesise that increasing protein intake in the morning may negatively impact total protein intake.

It has been suggested that 25–30 g of dietary protein per meal is required to maximally stimulate skeletal muscle protein synthesis and prevent sarcopenia^(29)^. This would imply that morning intake, which is 13·5 (se 0·3) g in our population, should be increased. The mean absolute protein intake in the morning was somewhat lower on days with a low protein intake compared to days with a high protein intake (11·4 *v*. 14·1 g, respectively). The differences in absolute protein intake mid-day (16·9 *v*. 28·9 g) and in the evening (22·6 *v*. 38·5 g) were much larger, resulting in a higher proportion of protein intake in the morning on days with a low protein intake. To improve protein intake, it may therefore be better to focus more on the hours after noon.

Drawing conclusions on the effect of timing of protein intake on total protein intake from our study is thus difficult. Therefore, experimental studies actively manipulating protein intakes at different hours of the day are needed to study the effect of timing of protein intake on total protein intake and to define optimal timing strategies to increase the likelihood of adequate protein intake. Such experimental studies will also shed more light on the possible clinical implications of manipulating the timing of protein intake.

Strengths of the present study include the use of exact timing of meals, enabling us to look more closely at the protein intake pattern compared to studies that only collected data on main meals and/or other food consumption occasions. Another strength of our study is the adjustment for wake-up time, which is a significant confounding factor, especially among older adults where a large variation in wake-up time exists^(30,31)^. Yet, none of the referenced studies addressed wake-up time. In the current study, wake-up time ranged from 00.00 to 11.00 hours, but did not differ between days with high and low protein intakes. In addition, both the regression coefficient and the OR for time of protein intake since wake-up were smaller compared to the estimates for the absolute time of protein intake. This suggests that absolute time is more important in this context than time since wake-up. The use of 24-h recalls combined with the food dairy is another strength, as it provided detailed information on specific foods consumed. The food diary was filled out at the time of consumption, thereby reducing reliance on memory and minimising the probability of misreporting by participants^(32)^.

A limitation of the present study is its cross-sectional design that did not allow making inferences on causal relations. In addition, the participation rate was low (26 %). Subjects who participated in DNFCS–Older Adults had lesser cognitive and physical impairments than the general population of older Dutch adults. Only few older adults with functional disabilities took part, and they had the lowest protein intake^(6)^. If the survey would have been more representative for the general older population, more older adults with disabilities must be included. This would probably have resulted in more days with a low protein intake and a lower average protein intake on these days.

To conclude, a larger *amount* of protein intake during morning, mid-day or evening hours is associated with a higher total protein intake among Dutch community-dwelling older adults. However, a higher *proportion* of protein in the morning was associated with a lower total protein intake, whereas the proportion of protein ingested mid-day or evening showed an opposite but weaker association with total protein intake. This suggests that the timing of protein intake may also be important. Future research should focus on establishing whether active manipulation of the timing of protein intake could influence total protein intake. If confirmed, this may have implications in dietary strategies to prevent protein-related health outcomes, such as PEM, among older adults.

## Supplementary material

For supplementary material accompanying this paper visit https://doi.org/10.1017/S1368980020000026. click here to view supplementary material

## References

[1] Wolfe RR (2012) The role of dietary protein in optimizing muscle mass, function and health outcomes in older individuals. Br J Nutr 108, Suppl. 2, S88–S93. 23107552 10.1017/S0007114512002590

[2] Houston DK , Tooze JA , Garcia K et al. (2017) Protein intake and mobility limitation in community-dwelling older adults: the health ABC study. J Am Geriatr Soc 65, 1705–1711. 28306154 10.1111/jgs.14856 PMC5555791

[3] Mendonça N , Granic A , Hill TR et al. (2019) Protein intake and disability trajectories in very old adults: the Newcastle 85+ Study. J Am Geriatr Soc 67, 50–56. 30382594 10.1111/jgs.15592 PMC6334273

[4] Hengeveld LM , Wijnhoven HAH , Olthof MR et al. (2018) Prospective associations of poor diet quality with long-term incidence of protein-energy malnutrition in community-dwelling older adults: the Health, Aging, and Body Composition (Health ABC) Study. Am J Clin Nutr 107, 155–164. 29529142 10.1093/ajcn/nqx020 PMC6248415

[5] European Food Safety Association (EFSA) (2017) *Dietary Reference Values for Nutrients: Summary Report*. EFSA supporting publication 2017, e15121.

[6] Ocké MC , Buurma-Rethans EJM , de Boer EJ et al. (2013) Diet of Community-Dwelling Older Adults. Dutch National Food Consumption Survey-Older Adults 2010–2012. RIVM report 050413001. Bilthoven: National Institute for Public Health and the Environment.

[7] Health Council of the Netherlands (2011) Undernutrition in the Elderly. Report no. 2011/32E. The Hague: Health Council of the Netherlands.

[8] Halfens RJG , Meesterberends E , Neyens JCL et al. (2015) Landelijke prevalentiemeting zorgproblemen: Rapportage resultaten 2015 [National Prevalence Measurement of Health Problems: Reporting Results 2015]. Maastricht: Universiteit Maastricht.

[9] Schilp J , Kruizenga HM , Wijnhoven HAH et al. (2012) High prevalence of undernutrition in Dutch community-dwelling older individuals. Nutrition 28, 1151–1156. 22749873 10.1016/j.nut.2012.02.016

[10] de Castro JM (2007) The time of day and the proportions of macronutrients eaten are related to total daily food intake. Br J Nutr 98, 1077–1083. 17537291 10.1017/S0007114507754296

[11] Mendonça N , Granic A , Mathers JC et al. (2018) Prevalence and determinants of low protein intake in very old adults: insights from the Newcastle 85+ Study. Eur J Nutr 57, 2713–2722. 28948346 10.1007/s00394-017-1537-5 PMC6267410

[12] Paddon-Jones D , Campbell WW , Jacques PF et al. (2015) Protein and healthy aging. Am J Clin Nutr 101, 1339S–1345S. 25926511 10.3945/ajcn.114.084061

[13] Tieland M , Borgonjen-Van den Berg KJ , van Loon LJ et al. (2012) Dietary protein intake in community-dwelling, frail, and institutionalized elderly people: scope for improvement. Eur J Nutr 51, 173–179. 21562887 10.1007/s00394-011-0203-6

[14] Huseinovic E , Winkvist A , Slimani N et al. (2016) Meal patterns across ten European countries – results from the European Prospective Investigation into Cancer and Nutrition (EPIC) calibration study. Public Health Nutr 19, 2769–2780. 27194183 10.1017/S1368980016001142 PMC10271196

[15] Bollwein J , Diekmann R , Kaiser MJ et al. (2013) Distribution but not amount of protein intake is associated with frailty: a cross-sectional investigation in the region of Nurnberg. Nutr J 12, 109. 23915061 10.1186/1475-2891-12-109 PMC3750269

[16] Arnal M-A , Mosoni L , Boirie Y et al. (1999) Protein pulse feeding improves protein retention in elderly women. Am J Clin Nutr 69, 1202–1208. 10357740 10.1093/ajcn/69.6.1202

[17] Leidy HJ , Bossingham MJ , Mattes RD et al. (2009) Increased dietary protein consumed at breakfast leads to an initial and sustained feeling of fullness during energy restriction compared to other meal times. Br J Nutr 101, 798–803. 19283886 10.1017/s0007114508051532

[18] Kim IY , Schutzler S , Schrader A et al. (2015) Quantity of dietary protein intake, but not pattern of intake, affects net protein balance primarily through differences in protein synthesis in older adults. Am J Physiol Endocrinol Metab 308, E21–E28. 25352437 10.1152/ajpendo.00382.2014 PMC4280213

[19] de Vries JH , de Groot LC & van Staveren WA (2009) Dietary assessment in elderly people: experiences gained from studies in the Netherlands. Eur J Clin Nutr 63, Suppl. 1, S69–S74. 19190649 10.1038/ejcn.2008.68

[20] Slimani N , Deharveng G , Charrondiere RU et al. (1999) Structure of the standardized computerized 24-h diet recall interview used as reference method in the 22 centers participating in the EPIC project. European Prospective Investigation into Cancer and Nutrition. Comput Methods Programs Biomed 58, 251–266. 10094230 10.1016/s0169-2607(98)00088-1

[21] Slimani N , Ferrari P , Ocke M et al. (2000) Standardization of the 24-hour diet recall calibration method used in the European prospective investigation into cancer and nutrition (EPIC): general concepts and preliminary results. Eur J Clin Nutr 54, 900–917. 11114689 10.1038/sj.ejcn.1601107

[22] Slimani N , Valsta L & EFCOSUM Group (2002) Perspectives of using the EPIC-SOFT programme in the context of pan-European nutritional monitoring surveys: methodological and practical implications. Eur J Clin Nutr 56, Suppl. 2, S63–S74. 12082519 10.1038/sj.ejcn.1601430

[23] NEVO-tabel (2011). Nederlands Voedingsstoffenbestand 2011 [Dutch Food Composition Database 2011]. Den Haag: RIVM/Voedingscentrum.

[24] Berner LA , Becker G , Wise M et al. (2013) Characterization of dietary protein among older adults in the United States: amount, animal sources, and meal patterns. J Acad Nutr Diet 113, 809–815. 23491327 10.1016/j.jand.2013.01.014

[25] Wijnhoven HA , Schilp J , van Bokhorst-de van der Schueren MA et al. (2012) Development and validation of criteria for determining undernutrition in community-dwelling older men and women: the short nutritional assessment questionnaire 65+. Clin Nutr 31, 351–358. 22119209 10.1016/j.clnu.2011.10.013 PMC6121713

[26] Wendel-Vos GC , Schuit AJ , Saris WH et al. (2003) Reproducibility and relative validity of the short questionnaire to assess health-enhancing physical activity. J Clin Epidemiol 56, 1163–1169. 14680666 10.1016/s0895-4356(03)00220-8

[27] Ocke MC , Larranaga N , Grioni S et al. (2009) Energy intake and sources of energy intake in the European Prospective Investigation into Cancer and Nutrition. Eur J Clin Nutr 63, Suppl. 4, S3–S15. 19888279 10.1038/ejcn.2009.72

[28] Haines PS , Hama MY , Guilkey DK et al. (2003) Weekend eating in the United States is linked with greater energy, fat, and alcohol intake. Obes Res 11, 945–949. 12917498 10.1038/oby.2003.130

[29] Paddon-Jones D & Rasmussen BB (2009) Dietary protein recommendations and the prevention of sarcopenia: protein, amino acid metabolism and therapy. Curr Opin Clin Nutr Metab Care 12, 86. 19057193 10.1097/MCO.0b013e32831cef8b PMC2760315

[30] Ohayon MM & Vecchierini MF (2005) Normative sleep data, cognitive function and daily living activities in older adults in the community. Sleep 28, 981–989. 16218081

[31] Reyner LA , Horne JA & Reyner A (1995) Gender- and age-related differences in sleep determined by home-recorded sleep logs and actimetry from 400 adults. Sleep 18, 127–134. 7792492

[32] Baranowski T (2012) 24-Hour recall and diet record methods. In Nutritional Epidemiology, 3rd ed., pp. 49–69 [ W Willet , editor]. New York: Oxford University Press.

