# Supplementary material for: Protein intake pattern over the day and its association with low total protein intake in Dutch community-dwelling older adults
Source: Public Health Nutr. 2020 May 15;24(6):1415–27. doi: 10.1017/S1368980020000026 (PMC8025114; doi:10.1017/S1368980020000026)
Supplement: Supplementary file 1 [file S1368980020000026sup001.docx]

**Protein intake patterns over the day and its association with low total protein intake in Dutch community-dwelling older adults - Supplementary material**


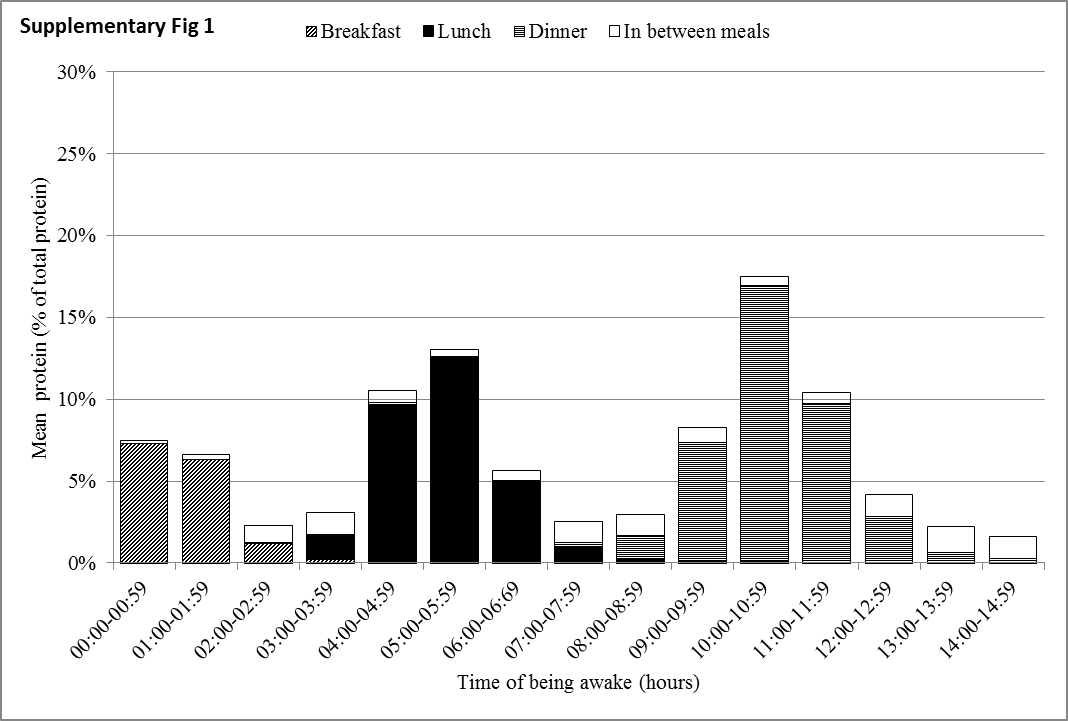


**Supplementary Figure 1: Proportion of total protein intake of Dutch community-dwelling older adults aged 70 years and older across time since wake-up and specified for breakfast, lunch, dinner and in between meals (DNFCS-Older adults 2010-2012).**
